# External applicability of SGLT2 inhibitor cardiovascular outcome trials to patients with type 2 diabetes and cardiovascular disease

**DOI:** 10.1186/s12933-021-01373-9

**Published:** 2021-09-08

**Authors:** Lisanne C. A. Smidt, Frank L. J. Visseren, Wendela L. de Ranitz-Greven, Hendrik M. Nathoe, L. Jaap Kappelle, Gert J. de Borst, Harold W. de Valk, Jan Westerink, the UCC-SMART Study Group

**Affiliations:** 1 Department of Vascular Medicine, University Medical Center Utrecht, Heidelberglaan 100, 3584 CX Utrecht, The Netherlands; 2 Department of Endocrinology, University Medical Center Utrecht, Utrecht, The Netherlands; 3 Department of Cardiology, University Medical Center Utrecht, Utrecht, The Netherlands; 4 Department of Neurology, University Medical Center Utrecht, Utrecht, The Netherlands; 5 Department of Vascular Surgery, University Medical Center Utrecht, Utrecht, The Netherlands

**Keywords:** Applicability, Cardiovascular disease, Cardiovascular outcome trial, Sodium-glucose cotransporter 2 inhibitor, Type 2 diabetes

## Abstract

**Background:**

Recent treatment guidelines support the use of sodium-glucose cotransporter 2 inhibitors (SGLT2i) in patients with type 2 diabetes and cardiovascular disease based on the results of cardiovascular outcome trials (CVOTs). Applicability of these trials to everyday patients with type 2 diabetes and cardiovascular disease is however unknown. The aim of this study is to assess the external applicability of SGLT2i CVOTs in daily clinical practice type 2 diabetes patients with established cardiovascular disease.

**Methods:**

Trial in- and exclusion criteria from EMPA-REG OUTCOME, CANVAS, DECLARE-TIMI 58 and VERTIS-CV were applied to 1389 type 2 diabetes patients with cardiovascular disease in the Utrecht Cardiovascular Cohort-Secondary Manifestations of ARTerial disease (UCC-SMART). To evaluate the difference in cardiovascular risk (MACE) and all-cause mortality between trial eligible and ineligible patients, age and sex-adjusted Cox-regression analyses were performed.

**Results:**

After applying trial in- and exclusion criteria, 48% of UCC-SMART patients with type 2 diabetes and cardiovascular disease would have been eligible for DECLARE-TIMI 58, 35% for CANVAS, 29% for EMPA-REG OUTCOME and 21% for VERTIS-CV. Without the eligibility criteria of HbA_1c_, eligibility was 58–88%. For all trials the observed risk for cardiovascular events and all-cause mortality was similar in eligible and ineligible patients after adjustment for age and gender.

**Conclusion:**

A large proportion of patients with type 2 diabetes and cardiovascular disease in daily clinical practice would have been eligible for participation in the SGLT2i CVOTs. Trial eligible and ineligible patients have the same risk for MACE and all-cause mortality.

**Supplementary Information:**

The online version contains supplementary material available at 10.1186/s12933-021-01373-9.

## Introduction

In the recent years, four cardiovascular outcome trials (CVOTs) have been conducted to assess the safety and efficacy of sodium-glucose cotransporter 2 inhibitors (SGLT2i) on cardiovascular end-points in high risk type 2 diabetes patients (EMPA-REG OUTCOME, DECLARE-TIMI 58, CANVAS and VERTIS-CV) [1–4]. Meta-analysis of the first three of these trials has shown a reduced risk for cardiovascular events and mortality, especially in type 2 diabetes patients with cardiovascular disease at baseline [5]. Based on these results the 2019 European Society of Cardiology guidelines on diabetes and cardiovascular diseases, developed in collaboration with the European Association for the study of Diabetes, propose to make SGLT2i a first line glucose lowering drug in patients with type 2 diabetes and cardiovascular disease [6].

Randomized clinical trials have strict in- and exclusion criteria in order to define a clearly delineated population to pursue internal validity. However, strict selection of participants upfront might impair the applicability of trial results to a more general population and may select (unknowingly) on patients with a better or worse a priori outcome [7, 8]. Benefits and harms from a treatment found in randomized clinical trials might therefore be an over- or underestimation of the real-world outcomes.

In order to quantify the applicability of SGLT2i CVOTs to real world patients, several studies have been conducted in which trial in- and exclusion have been applied to populations of type 2 diabetes patient both with and without cardiovascular disease [9–16]. Within these broad populations, the main criterion limiting trial applicability was selection on either pre-existing cardiovascular disease or presence of cardiovascular risk factors. Since the new treatment guidelines recommend the use of SGLT2i in patients with established cardiovascular disease, applicability within this subgroup is now specifically relevant. Furthermore, no data is currently available on whether eligible and ineligible real-world patients have a different prognosis.

Therefore, the objective of this paper is to assess the external applicability of guideline-informing SGLT2i CVOTs in a real-world cohort consisting of patients with type 2 diabetes and concomitant cardiovascular disease.

## Methods

### Study population

Data were used from patients enrolled in the Utrecht Cardiovascular Cohort-Secondary Manifestations of ARTerial disease Utrecht Cardiovascular Cohort (UCC-SMART). The UCC-SMART study is an ongoing prospective cohort study at the University Medical Center Utrecht, The Netherlands. From September 1996 onwards, newly referred patients with clinically manifest vascular disease, or important risk factors for atherosclerotic disease (diabetes, hyperlipidaemia, hypertension) were asked to participate. Written informed consent was obtained from all participants. The Medical Ethics Committee of the University Medical Center Utrecht approved the study. A detailed description of the study design has been published previously [17].

For the present study, data were used from 1389 patients with type 2 diabetes and established cardiovascular disease, included between September 1996 and February 2017. Type 2 diabetes was defined as either a referral or self-reported diagnosis of type 2 diabetes, or a fasting glucose of ≥ 7.0 mmol/L at baseline plus initiation of diabetes treatment within 1 year after inclusion or the use of glucose-lowering medication at inclusion. Patients with type 1 diabetes were excluded. Cardiovascular disease at baseline was defined as a either a diagnosis of coronary artery, cerebrovascular, peripheral artery disease or abdominal aortic aneurysm as referral diagnosis or a self-reported history of any of these on a standardized questionnaire.

### Baseline data collection

After inclusion, patients underwent a standardized vascular screening protocol consisting of a health questionnaire including medical history and risk factors, physical examination and laboratory testing.

### Outcome assessment

During follow-up patients were asked to fill out a standardized questionnaire about new vascular events twice a year. When a possible event was reported, hospital discharge letters, results of relevant laboratory and radiology examinations were also collected. Events were independently evaluated by three members of the UCC-SMART study endpoint committee. The primary outcome of interest for the current study was composite of major cardiovascular events (MACE): cardiovascular mortality, non-fatal myocardial infarction and non-fatal stroke. Secondary outcomes included all-cause mortality and the individual components of the composite primary outcome. Definitions of outcome events have been published previously [17]. Duration of follow-up was defined as the period between study inclusion and the first cardiovascular event, death, loss to follow-up or the predefined date of March 1st 2018. In the current cohort 101 (7.3%) patients were lost to follow-up.

### CVOT eligibility criteria

In order to assess trial eligibility, first the in- and exclusion criteria of CVOTs on the SGLT2 inhibitors Empagliflozin (EMPA-REG OUTCOME) [1, 18], Canagliflozin (CANVAS) [2, 19, 20], Dapagliflozin (DECLARE-TIMI 58) [3, 21] and Ertugliflozin (VERTIS-CV) [4, 22] were identified. Secondly, these in- and exclusion criteria where applied to all type 2 diabetes patients with cardiovascular disease in the UCC-SMART, to assess the eligibility of UCC-SMART patients for each trial. Due to extensive availability of baseline data of patients in the UCC-SMART, a wide variety of inclusion and exclusion criteria including HbA1c, age, cardiovascular history, blood pressure, eGFR, BMI but also alcohol abuse or triglyceride level could be used in the current analysis. A complete overview of in- and exclusion criteria that were used are provided in Additional file 1: Table S2.

### Data analyses

The number of patients in the UCC-SMART eligible or ineligible for participation in each trial was calculated. Differences in baseline characteristics between trial eligible and ineligible patients were calculated with an unpaired Student’s *t* test for continuous variables if normally distributed or Wilcoxon rank sum test in case of a skewed distribution. For categorical valuables confidence intervals of differences in baseline characteristics were calculated with the two-proportion Z-test, variables with > 2 categories were divided into multiple binary variables in order to calculate difference in proportions. A p-value for significant difference when considering all categories in the original variable was tested with a Pearson’s χ^2^ test. Cumulative incidence of MACE and all-cause mortality and individual MACE components for patients that were eligible and ineligible for each trial were plotted for the first 15 years of follow-up in UCC-SMART. The crude hazard ratio for 15 year risk on MACE, all-cause mortality and individual MACE components for trial eligible vs ineligible patients, as well as a hazard ratio adjusted for age and sex was calculated using a Cox proportional hazard model. The Cox proportional hazard assumption was checked by visual inspection of Schoenfeld residuals plots. A two-sided p-value < 0.05 was considered significant.

Randomly missing values for baseline data were completed by multiple imputation using the aregImpute function of the Hmisc package in R, that uses additive regression, bootstrapping and predictive mean matching. Data were imputed for self-reported history of stroke (n = 3, 0.2%), self-reported history of amputation (n = 3, 0.2%), self-reported history of myocardial infarction or heart attack (n = 1, 0.07%), smoking (n = 6, 0.4%), body mass index (n = 3, 0.2%), systolic blood pressure (n = 3, 0.2%), diastolic blood pressure (n = 9, 0.6%), HbA_1c_ (n = 120, 8.6%), creatinine (n = 6, 0.4%), albuminuria (n = 79, 5.7%), total cholesterol (n = 6, 0.4%), HDL cholesterol (n = 9, 0.6%), LDL cholesterol (n = 95, 6.8%), triglycerides (n = 8, 0.6%). A few trial exclusion criteria such as TSH levels (407 missing, 29.3%) and thiazolidinedione use (156 missing, 11.3%) data were not imputed, since data was not systematically available before a certain date and therefore not missing at random. If data regarding an exclusion criterion was missing, a patient was considered as not fulfilling this exclusion criterion and therefore remaining eligible.

All analyses were performed using R statistical software, version 4.0.3 (R Foundation for Statistical Computing, Vienna, Austria).

## Results

### Baseline characteristics UCC-SMART vs CVOTs

Baseline characteristics of 1389 type 2 diabetes patients with established cardiovascular disease from UCC-SMART and baseline characteristics of the four CVOTs are provided in Additional file 1: Table S1. In UCC-SMART the median duration of diabetes was 5 (IQR 1–11) years, with 54% of patients having a duration of diabetes of 5 years or less. Mean HbA_1c_ level in UCC-SMART was 52.9 ± 13.1 mmol/mol (7.0 ± 1.2%) compared to 65–67 mmol/mol (8.1–8.3%) in the CVOTs. Most patients in UCC-SMART had established coronary artery disease at baseline (68%), followed by cerebrovascular disease (28%), peripheral artery disease (20%). In the CVOTs cohorts 76–86% of patients with cardiovascular history had coronary artery disease, 19–23% had cerebrovascular disease and 15–32% had peripheral artery disease. Patients with abdominal aortic aneurysm were also included in UCC-SMART, but not in the CVOTs.

### Trial eligibility

**Fig. 1:**
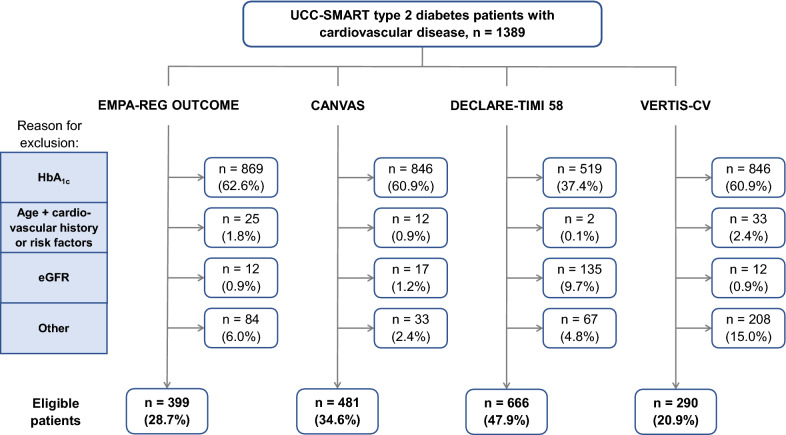
Flowchart of the process of applying the in- and exclusion criteria of cardiovascular outcome trials to UCC-SMART type 2 diabetes patients with cardiovascular disease

The proportion of UCC-SMART type 2 diabetes patients with established cardiovascular disease meeting CVOT eligibility criteria were 21% for VERTIS-CV, 29% for EMPA-REG OUTCOME, 35% for CANVAS and 48% for DECLARE-TIMI 58 (Fig. 1). There were 613 patients (44%) that were eligible for none of the trials and 224 patients (16%) fulfilled eligibility criteria for all four trials. Main reason for ineligibility for each trial was a HbA_1c_ below cut-off. In the UCC-SMART 823 patients (59%) had a HbA_1c_ below 53 mmol/mol (7%), which was the lower inclusion limit for EMPA-REG OUTCOME, VERTIS-CV and CANVAS and 512 patients (37%) had a HbA_1c_ below 48 mmol/mol (6.5%), which was the lower inclusion limit for DECLARE-TIMI 58. Without the HbA_1c_ criteria 58% (VERTIS-CV), 76% (DECLARE-TIMI 58), 80% (EMPA-REG OUTCOME) and 88% (CANVAS) of patients would have been eligible for participation in the SGLT2i CVOTs.

As DECLARE-TIMI 58 included patients with the broadest HbA_1c_ range of 48–108 mmol/mol (6.5–12%) most patients were eligible for this trial. DECLARE-TIMI 58 was more selective regarding eGFR limits than the other three CVOTs (exclusion if < 60 vs < 30 ml/min/1.73 m^2^). VERTIS-CV used a strict upper limit for blood pressure (160 mmHg systolic and 90 mmHg diastolic), which contributed to the least number of patients being eligible for this trial. DECLARE-TIMI 58 also had an upper limit for blood pressure, though less strict (180 mmHg systolic and 100 mmHg diastolic). The difference in eligibility between EMPA-REG OUTCOME and CANVAS is mainly based on the exclusion of patients with a blood glucose level of > 13.3 mmol/L at baseline in EMPA-REG OUTCOME, which was present in 6.6% of patients. A complete overview of the number of patients that were ineligible according to each criterion is provided in Additional file 1: Table S2.

### Differences in baseline characteristics between trial eligible vs ineligible patients

Table 1 shows differences in baseline characteristics between trial eligible and ineligible patients from the UCC-SMART cohort. An overview of the crude baseline characteristics of eligible and ineligible patients for each trial is provided in Additional file 1: Table S3. Patients eligible for DECLARE-TIMI 58 were younger than ineligible patients (61.6 ± 8.7 years vs 64.0 ± 8.6 years). There were more male among ineligible patients compared to eligible patients for EMPA-REG OUTCOME (78% vs 72%) and VERTIS-CV (77% vs 72%). As expected, most differences in baseline characteristics between eligible and ineligible patients reflect the in- and exclusion criteria of the trials. For example, for each CVOT the mean HbA_1c_ was higher amongst eligible patients compared to ineligible patients [HbA_1c_ difference: 12.1 mmol/mol (95% CI 11.0, 13.2) or 1.1% (95% CI 1.0, 1.2) for EMPA-REG OUTCOME; 16.2 mmol/mol (95% CI 15.1, 17.3) or 1.5% (95% CI 1.4, 1.6) for CANVAS; 12.1 mmol/mol (95% CI 10.9, 13.3) or 1.1% (95% CI 1.0, 1.2) for DECLARE-TIMI 58; and 11.9 mmol/mol (95% CI 10.6, 13.1) or 1.1% (95% CI 1.0, 1.2) for VERTIS-CV). Further, the eGFR of patients eligible for DECLARE-TIMI 58 was 11.7 ml/min/1.73 m^2^ (95% CI 9.7, 13.6) higher than the eGFR of ineligible patients. Finally, patients eligible for DECLARE-TIMI 58 and VERTIS-CV had a lower systolic and diastolic blood pressure than ineligible patients (DECLARE-TIMI 58 systolic − 5.8 mmHg (95% CI − 7.9, − 3.7), diastolic − 2.4 mmHg (95% CI − 3.6, − 1.4); VERTIS-CV systolic − 11.4 mmHg (95% CI − 13.5, − 9.4), diastolic − 5.9 mmHg (95% CI − 7.0, − 4.7)] (Table 1).

**Table 1:** Baseline differences between trial eligible and ineligible UCC-SMART type 2 diabetes patients with established cardiovascular disease

	Δ Eligible – ineligible (95% CI)			
	EMPA-REG OUTCOME	CANVAS	DECLARE-TIMI 58	VERTIS-CV
Patient characteristics				
Age (years)	− 0.2 (− 1.3, 0.8)	− 0.8 (− 1.8, 0.1)	**− 2.4 (− 3.4, − 1.5)**	− 1.0 (− 2.1, 0.2)
Male (%)	**− 5.5 (− 10.8, − 0.2)**	− 4.8 (− 9.7, 0.2)	− 0.1 (− 4.7, 4.5)	**− 5.3 (− 11.2, 0.7)**
Diabetes duration (years)	**1.5 (1.0, 2.5)**	**2.0 (1.0, 2.5)**	**1.0 (0.5, 2)**	**2.0 (1.0, 3.0)**
Smoking current (%)	3.3 (− 2.0, 8.6)	**5.7 (0.6, 10.7)**	**6.7 (2.0, 11.4)**	3.9 (− 2.1, 9.9)
History				
Coronary artery disease (%)	5.0 (− 0.5, 10.5)	1.7 (− 3.6, 7)	2.8 (− 2.2, 7.9)	5.3 (− 0.8, 11.4)
Cerebrovascular disease (%)	− 3.6 (− 8.9, 1.7)	− 3.6 (− 8.7, 1.4)	**− 6.3 (− 11.2, − 1.5)**	− 5.3 (− 11.2, 0.5)
Peripheral artery disease (%)	4.8 (− 0.2, 9.8)	**5.7 (1.0, 10.5)**	1.1 (− 3.3, 5.5)	4.2 (− 1.5, 9.8)
Medication				
Total glucose lowering therapy (%)	**12.7 (8.2, 17.2)**	**11.9 (7.5, 16.3)**	**9.1 (4.7, 13.6)**	**8.2 (3.0, 13.3)**
Glucose lowering agents (%)	3.1 (− 2.5, 8.8)	0.4 (− 5.0, 5.8)	3.2 (− 1.9, 8.4)	− 3.0 (− 9.4, 3.5)
Insulin (%)	**18.7 (13.2, 24.1)**	**20.0 (14.9, 25.1)**	**13.3 (8.7, 17.9)**	**19.5 (13.2, 25.8)**
Antihypertensive therapy (%)	2.7 (− 1.6, 7.0)	0.6 (− 3.6, 4.8)	− 3.3 (− 7.3, 0.7)	0.6 (− 4.3, 5.5)
Lipid lowering therapy (%)	0.4 (− 4.7, 5.6)	− 4.0 (− 9.0, 1.0)	− 1.2 (− 5.8, 3.5)	2.8 (− 2.9, 8.5)
Antithrombotic therapy (%)	1.6 (− 2.5, 5.6)	− 3.4 (− 7.4, 0.7)	− 2.3 (− 6.0, 1.5)	1.7 (− 2.8, 6.2)
Physical examination				
BMI (kg/m^2^)	0.3 (− 0.2, 0.8)	**0.6 (0.1, 1.1)**	**1.1 (0.7, 1.6)**	0.4 (− 0.2, 0.9)
Systolic BP (mmHg)	1.1 (− 1.3, 3.5)	1.6 (− 0.7, 3.9)	**− 5.8 (− 7.9, − 3.7)**	**− 11.4 (− 13.5, − 9.4)**
Diastolic BP (mmHg)	− 0.8 (− 2.1, 0.4)	− 0.2 (− 1.4, 1.1)	**− 2.4 (− 3.6, − 1.3)**	**− 5.9 (− 7.0, − 4.7)**
Laboratory results				
HbA1c (mmol/mol)	**12.1 (11.0, 13.2)**	**16.2 (15.1, 17.3)**	**12.1 (10.9, 13.3)**	**11.9 (10.6, 13.1)**
HbA1c (%)	**1.1 (1.0, 1.2)**	**1.5 (1.4, 1.6)**	**1.1 (1.0, 1.2)**	**1.1 (1.0, 1.2)**
eGFR (CKD-EPI) (ml/min/1.73 m^2^)	1.0 (− 1.2, 3.2)	**2.7 (0.6, 4.8)**	**11.7 (9.7, 13.6)**	1.4 (− 1.1, 3.9)
Total cholesterol (mmol/L)	0.0 (− 0.1, 0.2)	0.1 (0.0, 0.3)	− 0.1 (− 0.2, 0.03)	− 0.1 (− 0.2, 0.1)
HDL-C (mmol/L)	0.0 (− 0.1, 0.0)	**− 0.1 (− 0.1, 0.0)**	**0.03 (− 0.1, − 0.002)**	**− 0.04 (− 0.08, − 0.003)**
LDL-C (mmol/L)	0.0 (− 0.1, 0.1)	0.0 (− 0.1, 0.2)	**− 0.2 (− 0.3, − 0.1)**	− 0.04 (− 0.2, 0.1)
Triglycerides (mmol/L)	0.1 (0.0, 0.2)	**0.2 (0.1, 0.3)**	**0.1 (0.01, 0.2)**	0.1 (− 0.1, 0.2)

Data are presented as difference in proportion (%) for categorical variables, difference in median for duration of diabetes and triglycerides and difference in mean for other continues variables. Data in bold represent a p < 0.05 for the difference between eligible and ineligible patients. Information on baseline characteristics within the groups of trial eligible and trial ineligible patients are presented in Additional file 1: Table S3

*CKD-EPI* chronic kidney disease epidemiology collaboration formula, *HDL-C* high-density lipoprotein cholesterol, *LDL-C* low-density lipoprotein cholesterol

### Risk of MACE and all-cause mortality in eligible and ineligible patients

**Fig. 2:**
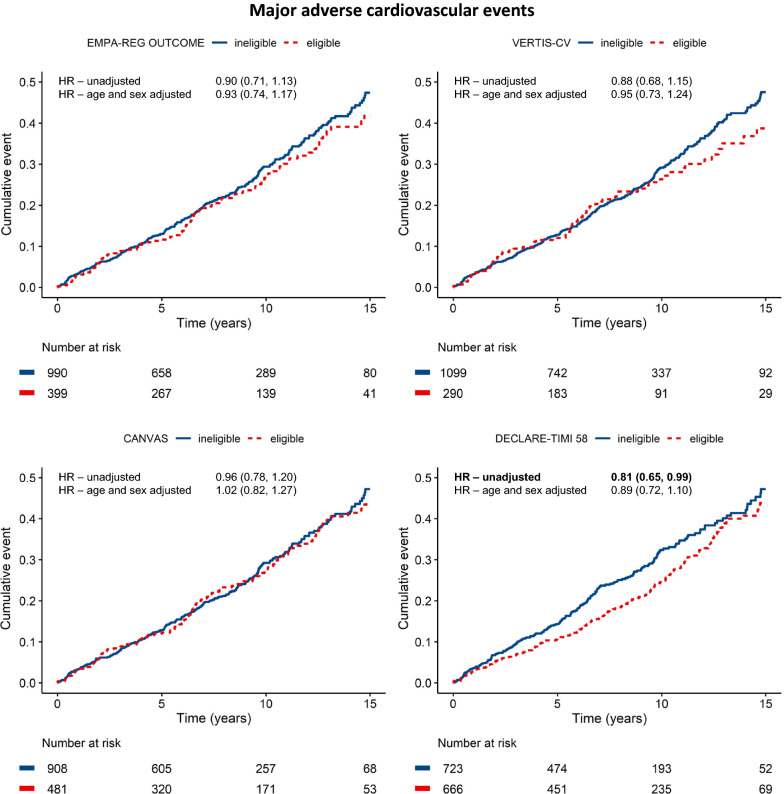
Major adverse cardiovascular events in trial eligible vs trial ineligible UCC-SMART type 2 diabetes patients with cardiovascular disease. Unadjusted hazard ratios and adjusted hazard ratios with 95% CI’s are presented for eligible vs ineligible patients for each trial. Data in bold represent a statistically significant difference between eligible and ineligible patients

**Fig. 3:**
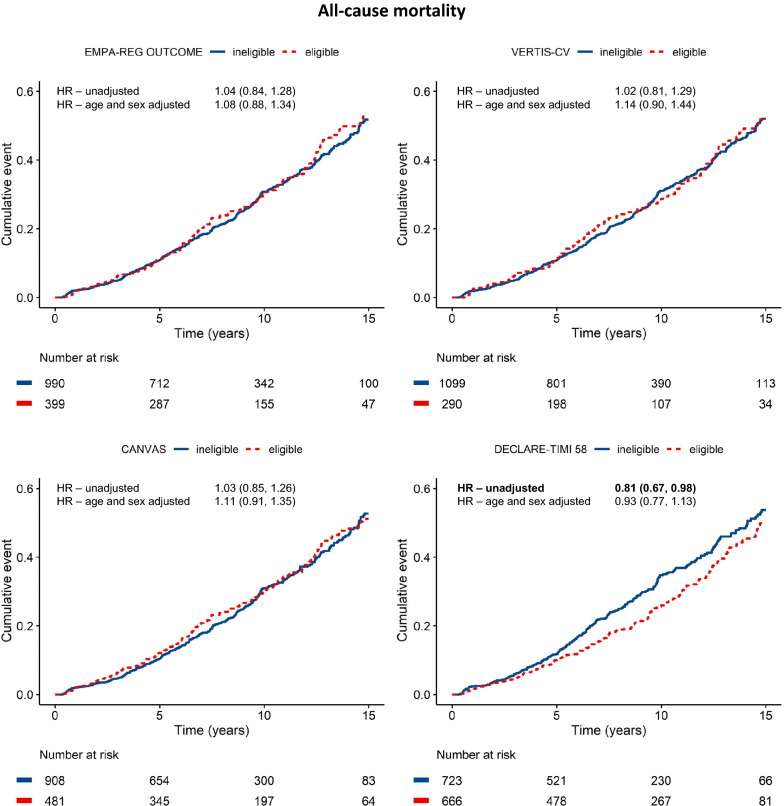
All-cause mortality in trial eligible vs trial ineligible UCC-SMART type 2 diabetes patients with cardiovascular disease. Unadjusted hazard ratios and adjusted hazard ratios with 95% CI’s are presented for eligible vs ineligible patients for each trial. Data in bold represent a statistically significant difference between eligible and ineligible patients

UCC-SMART type 2 diabetes patients were followed-up for a median of 8.14 years (IQR: 4.48–11.8). During follow-up, MACE occurred 377 times, all-cause mortality occurred 454 times. Figures 2 and 3 show cumulative incidence curves for MACE and all-cause mortality, divided by patients that would be eligible and ineligible for each trial, with unadjusted and adjusted hazard ratios for the 15-year risk on MACE and mortality. No significant differences in all-cause mortality or MACE between patients eligible and ineligible for EMPA-REG OUTCOME [HR 0.90 (95% CI 0.71, 1.13)], CANVAS [HR 0.96 (95% CI 0.78, 1.20)] and VERTIS-CV [HR 0.88 (95% CI 0.68, 1.15)] were found. Only for DECLARE-TIMI 58 there was a lower risk on MACE [HR 0.81 (95% CI 0.65, 0.99)] and mortality [HR 0.81 (95% CI 0.67, 0.98)] for eligible vs ineligible patients, however this did not remain statistically significant after adjustment for age and sex [MACE HR 0.89 (95% CI 0.72–1.10), mortality HR 0.93 (95% CI 0.77, 1.13)] Also, no statistically significant differences were found in individual MACE components between eligible and ineligible patients for all four CVOTs (Additional file 1: Table S4).

## Discussion

A large proportion of real-life patients with type 2 diabetes and cardiovascular disease would have been eligible for participation in the SGLT2i CVOTs (48% for DECLARE-TIMI 58, 35% for CANVAS, 29% for EMPA-REG OUTCOME, 21% for VERTIS-CV). Most important reason for ineligibility was a HbA_1c_ below cut-off per trial (37% for DECLARE-TIMI 58, 59% for the other CVOTS). In the absence of the HbA_1c_ selection criteria, the majority of patients would have been eligible (58–88%). The eligible and ineligible patients in the UCC-SMART had a similar observed risk for cardiovascular disease and all-cause mortality. These results make it unlikely that the selection criteria from the SGLT2i cardiovascular outcome trials hamper their applicability to the broad population of patients with type 2 diabetes and cardiovascular disease at baseline. These results therefore may aid the implementation of new guidelines suggesting SGLT2i as the first line treatment for patients with type 2 diabetes and prior cardiovascular disease.

This study specifically focused on eligibility in the subgroup of type 2 diabetes patients with established cardiovascular disease as this is currently one of the most relevant target populations for SGLT2i use. The most recent guideline of the European Society of Cardiology and the European Association for the Study of Diabetes on diabetes and cardiovascular disease currently recommends the use of SGLT2i as a first-line antihyperglycemic agent in this patient group [6]. Earlier applicability studies have assessed eligibility for SGLT2i CVOT participation in large cohorts of type 2 diabetes patients both with and without cardiovascular disease [9–16]. A systematic review and meta-analysis of five of these earlier applicability studies reported eligibility percentages that were comparable to the eligibility found in the present study (17–49%).[23] However, there are large differences between the individual studies. Wittbrodt et al. reported an eligibility of 26–27% for EMPA-REG OUTCOME and VERTIS-CV in patients included in the diabetes collaborative registry in the United States [10], whereas Pintat et al. and Nicolucci et al. reported an eligibility of only 7–8% for these studies [11, 13]. These differences depend largely on the amount of patients with cardiovascular disease that vary from 14 to 64% in the different earlier cohorts. Eligibility for DECLARE-TIMI 58 was more comparable across different cohorts. Consequently, in cohorts consisting of fewer patients with cardiovascular disease, the differences in eligibility for EMPA-REG OUTCOME or VERTIS-CV (patients with cardiovascular disease) and for DECLARE-TIMI 58 (patients with ≥ 1 cardiovascular risk factor) were larger than in cohorts consisting of more patients with cardiovascular disease and especially larger than in the current study consisting of only patients with a cardiovascular history. Although the numbers of patients included in the current analysis are smaller than included in earlier applicability studies, the current analysis provides more specific information about eligibility in the subgroup of patients with both type 2 diabetes and cardiovascular disease. Another difference between the current study and earlier applicability studies is that more baseline data are used for eligibility assessment in the current study. Overall, the extensive use of data for analysis, thus the possibility to incorporate more of the trials in and- exclusion criteria into the eligibility assessments, leads to a slightly lower eligibility than when only data on HbA1c, cardiovascular history, eGFR and BMI would have been incorporated in analysis as was the case in most earlier applicability studies. The availability of more extensive baseline data for assessing eligibility also explains the difference between eligibility for EMPA-REG OUTCOME and VERTIS-CV in the present study (in which eligibility for EMPA-REG OUTCOME is higher) versus earlier eligibility studies (in which eligibility for VERTIS-CV is often slightly higher). In contrast to earlier eligibility studies, we also incorporated baseline blood pressure into eligibility assessment, which lead to exclusion of patients for VERTIS-CV, but not for EMPA-REG OUTCOME that did not have a blood pressure limit.

The current study distinguishes itself from earlier applicability studies by assessing the risk on MACE and all-cause mortality in patients with type 2 diabetes and concomitant cardiovascular disease eligible and ineligible for SGLT2i CVOT participation based long-term cohort follow-up data. This makes it possible to evaluate whether inclusion and exclusion criteria at baseline not only select on these characteristics, but also select a particularly high or low risk subpopulation that would make extrapolation of the CVOT results to a larger population difficult. The current analysis does not raise major concerns regarding applicability of SGLT2i CVOT results to a larger population of type 2 diabetes patients with cardiovascular disease, as the only differences in outcome data between eligible and ineligible patients—a lower risk on MACE and all-cause mortality for patients eligible for DECLARE-TIMI 58—did not remain significant after adjustment for age and sex.

The main reason for ineligibility for SGLT2i CVOT in the current analysis was HbA_1c_ level for all four trials, as 59% of patients in our cohort had a HbA_1c_ below 53 mmol/mol (7%) (VERTIS-CV, EMPA-REG OUTCOME, CANVAS) and 37% below 48 mmol/mol (6.5%) (DECLARE-TIMI 58). Treatment with SGLT2i however shouldn’t necessarily be limited to patients above a certain HbA_1c_ target level. Even though exact mechanism of the positive effect of SGLT2i on cardiovascular outcomes has not been entirely clarified, it is known that this positive effects is irrespective of the antihyperglycemic effect [24, 25].

Strengths of this study include the large prospective cohort of patients with diabetes and cardiovascular disease seen in daily clinical practice, the extensiveness of collected patient characteristics, the long follow-up and the completeness of data. Extensive availability of baseline data made it possible to assess eligibility on a large part of the CVOTs in- and exclusion criteria. The availability of long-term follow-up data made it possible to assess differences in outcomes between eligible and ineligible patients. A potential limitation to this study is that our cohort consist of patients with a relatively short duration of diabetes and lower use of antihyperglycemic agents, and lower HbA_1c_ level compared to the CVOTs. However, a cohort with patients with more progressed diabetes would have only led to a higher eligibility percentage, because of fewer patients being ineligible because of a HbA_1c_ level below cut-off. Another potential limitation is that the UCC-SMART cohort consists of patients that consented to participate. This might have induced selection bias, as elderly people with multimorbidity more often decline to invest in participating in a long-term follow-up study, leading to unintentionally selecting low-risk patients. On the other hand, selection bias towards more high-risk patients could have occurred, as the UCC-SMART consists of patients that have been referred to an university medical centre. Therefore the current analysis may not cover all type 2 diabetes patients with cardiovascular disease, but does provide insight in trial applicability to type 2 diabetes patients with cardiovascular disease referred to a hospital. Finally, as no data was available for heart failure at baseline or as an outcome measure, we could not assess the applicability of DAPA-HF and EMPEROR-Reduced [26, 27].

Future studies on real-world of applicability SGLT2i CVOTS should consist of larger (international) cohorts of type 2 diabetes patients, that also have follow-up data available to calculate the risk on adverse (cardiovascular) outcome for trial eligible and ineligible patients. Future studies could also focus on the primary care population and assess eligibility for both type 2 diabetes patients with cardiovascular disease and type 2 diabetes patients with only cardiovascular risk factors as separate groups. Applicability studies of SGLT2i heart-failure trials to real-world heart-failure patients, could provide more insight into external applicability of trials in this patient group.

## Conclusions

A large proportion of patients with type 2 diabetes and cardiovascular disease in daily clinical practice would have been eligible for participation in the SGLT2-inhibitor CVOTs. Main reason for trial ineligibility is the level of HbA_1c_. Trial eligible and ineligible patients have the same risk for MACE and all-cause mortality. Based on the current analysis, trial selection criteria are unlikely to confine the applicability of SGLT2i CVOT results to a larger population of type 2 diabetes patients with established cardiovascular disease.

## Supplementary Information

**Additional file 1: Table S1.** Baseline characteristics UCC-SMART type 2 diabetes patients with cardiovascular history and SGLT2 inhibitor cardiovascular outcome trials. **Table S2.** Number of UCC-SMART patients ineligible per criterion. **Table S3.** Baseline characteristics trial eligible and trial  ineligible UCC-SMART patients. **Table S4.** Hazard ratios for individual MACE components in trial eligible vs ineligible patients.

## Data Availability

The data that support the findings of this study are available from the corresponding author upon reasonable request.

## Abbreviations

CANVAS: Canagliflozin cardiovascular assessment study
CVOT: Cardiovascular outcome trial
DECLARE–TIMI 58: Dapagliflozin effect on cardiovascular events–thrombolysis in myocardial infarction
EMPA-REG OUTCOME: Empagliflozin cardiovascular outcome event trial in type 2 diabetes mellitus patients-removing excess glucose
MACE: Major adverse cardiovascular events
SGLT2i: Sodium-glucose cotransporter 2 inhibitor
UCC-SMART: Utrecht Cardiovascular Cohort-Secondary Manifestations of ARTerial disease
VERTIS-CV: Evaluation of ertugliflozin efficacy and safety cardiovascular outcomes
