# Supplementary material for: External applicability of SGLT2 inhibitor cardiovascular outcome trials to patients with type 2 diabetes and cardiovascular disease
Source: Cardiovasc Diabetol. 2021 Sep 8;20:181. doi: 10.1186/s12933-021-01373-9 (PMC8427950; doi:10.1186/s12933-021-01373-9)
Supplement: Supplementary file 1 — Additional file 1: Table S1. Baseline characteristics UCC-SMART type 2 diabetes patients with cardiovascular history and SGLT2 inhibitor cardiovascular outcome trials. Table S2. Number of UCC-SMART patients ineligible per criterion. Table S3. Baseline characteristics trial eligible and trial ineligible UCC-SMART patients. Table S4. Hazard ratios for individual MACE components in trial eligible vs ineligible patients. [file 12933_2021_1373_MOESM1_ESM.docx]

**Additional file 1**

Content

- Table S1: Baseline characteristics UCC-SMART type 2 diabetes patients with cardiovascular history and SGLT2 inhibitor cardiovascular outcome trials
- Table S2: Number of UCC-SMART patients ineligible per criterion
- Table S3: Baseline characteristics trial eligible and trial ineligible UCC-SMART patients
- Table S4: Hazard ratios for individual MACE components in trial eligible vs ineligible patients

**Table S1: Baseline characteristics UCC-SMART type 2 diabetes patients with cardiovascular history and SGLT2 inhibitor cardiovascular outcome trials**

|  | **UCC-SMART** | **EMPA-REG OUTCOME** | **CANVAS** | **DECLARE** | **VERTIS-CV** |
| --- | --- | --- | --- | --- | --- |
| **Number of patients** | 1389 | 7020 | 10142 | 17160 | 8238 |
| **Patient characteristics** |  |  |  |  |  |
| Age - years | 62.9 ± 8.8 | 63.1 ± 8.6 | 63.3 ± 8.3 | 63.9 ± 6.8 | 64.4 ± 8.1 |
| Male - % | 75.9 | 71.5 | 64.2 | 62.6 | 70.0 |
| Duration of diabetes - years | 5 (1 - 11) |  | 13.5 ± 7.8 | 11.0 (6.0 - 16.0) | 12.9 ± 8.3 |
| ≤5 - % | 53.8 | 18.0 |  | 22.4 |  |
| >5 to ≤10 - % | 20.8 | 24.9 |  | 27.6 |  |
| >10 to ≤15 - % | 11.7 | 57.1 |  | 23.0 |  |
| >15 to ≤20 - % | 7.1 |  |  | 14.2 |  |
| >20 - % | 6.6 |  |  | 12.9 |  |
| Current smoking - % | 25.3 | 13.2 | 17.8 | 14.5 |  |
| Former smoking - % | 53.6 | 45.7 |  |  |  |
| **History** |  |  |  |  |  |
| Cardiovascular history - % | 100.0 | 99.2 | 65.6 | 40.6 | 99.9 |
| Coronary artery disease - % | 67.9 | 75.6 | 56.4 | 33.0 | 76.3 |
| Cerebrovascular disease - % | 28.4 | 23.0 | 19.3 | 7.6 | 23.1 |
| Peripheral artery disease - % | 20.2 | 20.8 | 20.8 | 6.0 | 18.8 |
| AAA - % | 7.4 |  |  |  |  |
| **Medication** |  |  |  |  |  |
| Total glucose lowering  therapy - % | 77.7 |  |  |  |  |
| Glucose lowering agents - % | 65.4 |  |  |  |  |
| Metformin - % | 40.2† | 74.0 | 77.2 | 82.0 | 76.3 |
| Sulfonylurea - % | 23.1† | 42.8 | 43.0 | 42.7 | 41.1 |
| DPP-4 inhibitor - % | 1.4† | 11.3 | 12.4 | 16.8 | 11.0 |
| GLP-1 analogue - % | 0.4† | 2.8 | 4.0 | 4.4 | 3.4 |
| Thiazolidinedione- % | 2.3† | 4.3 |  |  | 1.9 |
| Insulin - % | 23.5 | 48.2 | 50.2 | 40.9 | 47.2 |
| Antihypertensive therapy - % | 84.0 |  |  |  |  |
| ACE inhibitor / ARB - % | 58.5 | 80.7 | 80.0 | 81.3 | 81.4 |
| Beta blocker - % | 57.9 | 64.9 | 53.5 | 52.6 | 69.1 |
| Diuretic - % | 34.6 | 14.1 | 44.3 | 40.6 | 40.6 |
| Calcium channel blocker - % | 26.7 | 33.0 |  | 33.0 |  |
| Lipid lowering therapy - % | 75.4 |  |  |  |  |
| Statin - % | 80.8^‡^ | 77.0 | 74.9 | 75.0 | 81.4 |
| Ezetimibe - % | 5.8^‡^ | 3.8 |  |  | 3.6 |
| Fibrates - % | 3.0^‡^ | 9.0 |  | 8.3 |  |
| Antithrombotic therapy - % | 86.6 |  | 73.6 |  |  |
| Antiplatelet - % | 78.0 |  |  | 61.1 | 84.6 |
| Acetylsalicylic acid - % | 73.8^‡^ | 82.7 |  | 52.1 |  |
| Clopidogrel - % | 32.1^‡^ | 10.6 |  | 10.9 |  |
| Oral anticoagulants - % | 13.9 |  |  |  |  |
| **Physical examination** |  |  |  |  |  |
| BMI - kg/m^2^ | 28.4 ± 4.3 | 30.6 ± 5.3 | 32.0 ± 5.9 | 32.1 ± 6.0 | 32.0 ± 5.4 |
| Systolic BP – mmHg | 144.4 ± 20.5 | 135.5 ± 17.0 | 136.6 ± 15.8 | 135.0 ± 15.4 | 133 ± 13.8 |
| Diastolic BP– mmHg | 80.8 ± 11.2 | 76.7 ± 9.8 | 77.7 ± 9.7 | 78.0 ± 9.1 | 77 ± 8.5 |
| **Laboratory results** |  |  |  |  |  |
| HbA1c – % | 7.0 ± 1.2 | 8.1 ± 0.8 | 8.2 ± 0.9 | 8.3 ± 1.2 | 8.3 ± 0.9 |
| eGFR – ml/min/1.73 m^2^ | 74.6 ± 19.6 | 74 ± 21 | 76.5 ± 20.5 | 85.3 ± 15.9 | 76.0 ± 20.9 |
| ≥90 | 23.7 | 22.0 | 24.4 | 48.0 | 24.8 |
| 60 to 90 - % | 54.9 | 52.0 | 55.5 | 45.0 | 53.3 |
| 30 to 60 - % | 19.7 | 26.0 | 19.8 | 7.0 | 21.6 |
| 15 to 30 - % | 1.5 |  | 0.3 |  | 0.4 |
| <15 - % | 0.2 |  | <0.1 |  |  |
| Albuminuria |  |  |  |  |  |
| Normal - % | 75.1 | 59.4 | 69.8 | 67.9 | 57.8 |
| Moderately increased  (ACR 3-30 mg/mmol) - % | 21.3 | 28.7 | 22.6 | 23.4 | 30.2 |
| Severely increased  (ACR ≥30 mg/mmol) - % | 3.7 | 11.0 | 7.6 | 6.8 | 9.2 |
| Total cholesterol – mmol/L | 4.6 ± 1.2 | 4.2 ± 1.1 | 4.4 ± 1.2 | 4.4 ± 1.2 | 4.4 ± 1.2 |
| HDL-C – mmol/L | 1.1 ± 0.3 | 1.1 ± 0.3 | 1.2 ± 0.3 | 1.2 ± 0.3 | 1.1 ± 0.3 |
| LDL-C – mmol/L | 2.6 ± 1.0 | 2.2 ± 0.9 | 2.3 ± 0.9 | 2.3 ± 0.9 | 2.3 ± 1.0 |
| Triglycerides – mmol/L | 1.6 (1.2 - 2.4) | 1.9 ± 1.4 | 2.0 ± 1.4 | 2.0 ± 1.5 | 2.0 ± 1.3 |

Legend text: Data are presented as percentage of total, mean ± standard deviation or median (interquartile range). †Based on 1106 patients included after July 2001. ‡Based on 1112 patients included after July 2001. Abbreviations: AAA: abdominal aortic aneurysm; DPP-4: dipeptidyl peptidase-4; GLP-1: glucagon-like peptide 1; ACE: angiotensin converting enzyme; ARB: angiotensin receptor blocker; ACR: albumin creatinine ratio; HDL-C: high-density lipoprotein cholesterol; LDL-C: low-density lipoprotein cholesterol

**Table S2: Number of UCC-SMART patients ineligible per criterion**

|  | **EMPA-REG OUTCOME** | | **CANVAS** | | **DECLARE-TIMI 58** | | **VERTIS-CV** | |
| --- | --- | --- | --- | --- | --- | --- | --- | --- |
|  | Criterion | Ineligible patients, N (%) | Criterion | Ineligible patients, N (%) | Criterion | Ineligible patients, N (%) | Criterion | Ineligible patients, N (%) |
| HbA1c | No glucose lowering therapy and HbA1c 7.0 – 9.0 %; or on glucose lowering therapy and HbA1c 7.0 – 10.0% | 869 (62.6%) | HbA1c 7.0 – 10.5% | 846 (60.9%) | HbA1c 6.5 - 12.0 % | 519 (37.4%) | HbA1c 7.0 – 10.5% | 846 (60.9%) |
| Age and CV history or risk factors† | ≥18 years and CV history | 86 (6.2%) | ≥30 with CV history; or ≥50 with ≥2 CV risk factors | 43 (3.1%) | ≥40 with CV history; or ≥55 (men) or ≥60 (women) with ≥1 CV risk factors | 3 (0.2%) | ≥40 with CV history | 99 (7.1%) |
| Blood pressure upper limit | - |  | - |  | SBP >180 or DBP >100 mmHg | 108 (7.8%) | SBP >160 or DBP >90 mmHg | 382 (27.5%) |
| eGFR | MDRD ≥ 30 ml/min/1.73m2 | 19 (1.4%) | CKD-EPI < 30 ml/min/1.73m2 | 24 (1.7%) | Cockroft-Gault < 60 ml/min | 213 (15.3%) | MDRD < 30 ml/min/1.73m2 | 19 (1.4%) |
| Blood glucose | >13.3 mmol/L | 92 (6.6%) | - |  | - |  | >15 mmol/L | 45 (3.2%) |
| Alcohol abuse | Alcohol intake ≥21 units per week; | 91 (6.6%) | Alcohol intake ≥21 units per week; | 91 (6.6%) | - |  | Alcohol intake ≥21 units per week; | 91 (6.6%) |
| Uncontrolled thyroid disorder | TSH <0.04 or >10.0 mU/L | 11 (0.8%) | TSH <0.04 or >10.0 mU/L | 11 (0.8%) | - |  | TSH <0.04 or >10.0 mU/L | 11 (0.8%) |
| Triglycerides | - |  | - |  | - |  | >6.78 mmol/L | 17 (1.2%) |
| Thiazolidinedione use | - |  | - |  | Thiazolidinedione use | 25 (1.8%) | Thiazolidinedione use | 25 (1.8%) |
| BMI | > 45 kg/m2; | 4 (0.3%) | - |  | - |  | ≥ 18.0 kg/m2; | 1 (0.1%) |
| HIV | - |  | Known history of HIV | 4 (0.3%) | - |  | Known history of HIV | 4 (0.3%) |
| Hemoglobin | - |  | - |  | - |  | <6.21 mmol/l | 13 (0.9%) |
| Total number of eligible patients | 391 (28.1%) | | 475 (34.2%) | | 667 (48%) | | 286 (20.6%) | |
|  | Inclusion criterion |  |  | Exclusion criterion | | - | No in- or exclusion criterion. | |

Legend text: † Cardiovascular history is defined slightly different in the different CVOTs and in the UCC-SMART therefore still some patients with CV history according to the UCC-SMART definition, are ineligible for the trials. Abbreviations: CV = cardiovascular; MDRD = Modification of Diet in Renal Disease formula; CKD-EPI = Chronic Kidney Disease Epidemiology Collaboration formula; TSH = thyroid stimulating hormone; HIV = human immunodeficiency virus.

**Table S3: Baseline characteristics trial eligible and trial ineligible UCC-SMART patients**

|  | **EMPA-REG OUTCOME** | | **CANVAS** | | **DECLARE-TIMI 58** | | **VERTIS-CV** | |
| --- | --- | --- | --- | --- | --- | --- | --- | --- |
|  | Eligible | Ineligible | Eligible | Ineligible | Eligible | Ineligible | Eligible | Ineligible |
| **Number of patients** | 399 | 990 | 481 | 908 | 666 | 723 | 290 | 1099 |
| **Patient characteristics** |  |  |  |  |  |  |  |  |
| Age - years | 62.7 ± 8.8 | 62.9 ± 8.8 | 62.3 ± 8.8 | 63.2 ± 8.7 | **61.6 ± 8.7** | **64 ± 8.6** | 62.1 ± 8.7 | 63.1 ± 8.8 |
| Male - % | **71.9** | **77.5** | 72.8 | 77.5 | 75.8 | 75.9 | **71.7** | **77** |
| Diabetes duration –  years | **7 (2 - 13)** | **4 (0.5 - 10)** | **7 (2 - 13)** | **4 (0.5 - 10)** | **6 (1 - 12)** | **4 (0.5 - 9)** | **7 (2 - 13.8)** | **4 (1 - 10)** |
| Smoking current - % | 27.6 | 24.2 | **28.9** | **23.2** | **28.7** | **22.0** | 28.3 | 24.4 |
| **History** |  |  |  |  |  |  |  |  |
| Coronary artery  disease - % | 71.4 | 66.5 | 69.0 | 67.3 | 69.4 | 66.5 | 72.1 | 66.8 |
| Cerebrovascular  disease - % | 25.8 | 29.4 | 26.0 | 29.6 | 25.1 | 31.4 | 24.1 | 29.5 |
| Peripheral artery  disease - % | 23.6 | 18.8 | 23.9 | 18.2 | **20.7** | **19.6** | 23.4 | 19.3 |
| **Medication** |  |  |  |  |  |  |  |  |
| Total glucose lowering  therapy - % | **86.7** | **74.0** | **85.4** | **73.6** | **82.4** | **73.3** | **84.1** | **76.0** |
| Glucose lowering  agents - % | 67.7 | 64.5 | 65.7 | 65.3 | 67.1 | 63.9 | 63.1 | 66.1 |
| Insulin - % | **36.8** | **18.2** | **36.6** | **16.6** | **30.5** | **17.2** | **39.0** | **19.5** |
| Antihypertensive  therapy - % | 86.0 | 83.2 | 84.4 | 83.8 | 82.3 | 85.6 | 84.5 | 83.9 |
| Lipid lowering  therapy - % | 75.7 | 75.3 | 72.8 | 76.8 | 74.8 | 75.9 | 77.6 | 74.8 |
| Antithrombotic  therapy - % | 87.7 | 86.2 | 84.4 | 87.8 | 85.4 | 87.7 | 87.9 | 86.3 |
| **Physical examination** |  |  |  |  |  |  |  |  |
| BMI - kg/m^2^ | 28.7 ± 4.3 | 28.4 ± 4.4 | **28.8 ± 4.5** | **28.3 ± 4.2** | **29.1 ± 4.4** | **27.9 ± 4.1** | 28.7 ± 4.5 | 28.4 ± 4.3 |
| Systolic BP – mmHg | 145.1 ± 20.8 | 143.9 ± 20.5 | 145.3 ± 21.1 | 143.7 ± 20.3 | **141.2 ± 17.1** | **147.1 ± 23** | **135.2 ± 14.3** | **146.7 ± 21.3** |
| Diastolic BP – mmHg | 80.2 ± 10.9 | 81.1 ± 11.4 | 80.7 ± 11.1 | 80.9 ± 11.4 | **79.6 ± 9.3** | **82 ± 12.7** | **76.2 ± 7.9** | **82.1 ± 11.7** |
| **Laboratory results** |  |  |  |  |  |  |  |  |
| HbA1c - % | **7.8 ± 0.7** | **6.7 ± 1.2** | **8.0 ± 0.8** | **6.5 ± 1.0** | **7.6 ± 1.0** | **6.5 ± 1.1** | **7.9 ± 0.8** | **6.8 ± 1.2** |
| HbA1c - mmol/mol | **61.6 ± 7.5** | **49.5 ± 13.3** | **63.5 ± 9.1** | **47.3 ± 11.3** | **59.3 ± 11.4** | **47.1 ± 11.8** | **62.3 ± 8.4** | **50.5 ± 13.0** |
| eGFR (CKD-EPI) –  ml/min/1.73 m^2^ | 75.4 ± 18.5 | 74.4 ± 20 | **76.4 ± 18.4** | **73.7 ± 20.1** | **80.8 ± 15.5** | **69.1 ± 21.2** | 75.8 ± 19.0 | 74.4 ± 19.7 |
| Total cholesterol –  mmol/L | 4.6 ± 1.2 | 4.6 ± 1.2 | 4.7 ± 1.2 | 4.5 ± 1.2 | 4.5 ± 1.2 | 4.6 ± 1.1 | 4.5 ± 1.2 | 4.6 ± 1.2 |
| HDL-C – mmol/L | 1.1 ± 0.3 | 1.1 ± 0.3 | **1.1 ± 0.3** | **1.1 ± 0.3** | **1.1 ± 0.3** | **1.1 ± 0.3** | **1.1 ± 0.3** | **1.1 ± 0.3** |
| LDL-C – mmol/L | 2.6 ± 1 | 2.6 ± 1.0 | 2.6 ± 1.0 | 2.6 ± 1.0 | **2.5 ± 1.0** | **2.7 ± 1.0** | 2.6 ± 1.0 | 2.6 ± 1.0 |
| Triglycerides – mmol/L | 1.7 (1.2 - 2.6) | 1.6 (1.2 - 2.4) | **1.8 (1.2 - 2.7)** | **1.6 (1.1 - 2.2)** | **1.7 (1.2 - 2.6)** | **1.6 (1.2 - 2.3)** | 1.7 (1.2 - 2.5) | 1.6 (1.2 - 2.4) |

Legend text: Data are presented as percentage of total, mean ± standard deviation or median (interquartile range). Data in bold represent a p<0.05 for the difference between eligible and ineligible patients. Abbreviations: CKD-EPI = Chronic Kidney Disease Epidemiology Collaboration formula; HDL-C = high-density lipoprotein cholesterol; LDL-C = low-density lipoprotein cholesterol.

**Table S4: Hazard ratios for individual MACE components in trial eligible vs ineligible patients**

|  |  | **EMPA-REG OUTCOME** | **CANVAS** | **DECLARE-TIMI 58** | **VERTIS-CV** |
| --- | --- | --- | --- | --- | --- |
| **HR (95% CI)** |  | Death from cardiovascular causes | | | |
|  | Unadjusted | 0.91 (0.68, 1.21) | 0.94 (0.72, 1.24) | 0.78 (0.60, 1.01) | 0.88 (0.63, 1.22) |
|  | Adjusted for age and sex | 0.95 (0.71, 1.27) | 1.02 (0.78, 1.33) | 0.91 (0.70, 1.18) | 0.98 (0.70, 1.37) |
|  |  | Non-fatal myocardial infarction | | | |
|  | Unadjusted | 0.91 (0.61, 1.37) | 0.93 (0.64, 1.35) | 0.74 (0.52, 1.07) | 0.83 (0.52, 1.33) |
|  | Adjusted for age and sex | 0.91 (0.61, 1.36) | 0.93 (0.64, 1.36) | 0.77 (0.53, 1.11) | 0.84 (0.53, 1.35) |
|  |  | Non-fatal stroke | | | |
|  | Unadjusted | 1.08 (0.66, 1.77) | 1.15 (0.72, 1.83) | 0.91 (0.58, 1.44) | 1.26 (0.74, 2.14) |
|  | Adjusted for age and sex | 1.10 (0.67, 1.80) | 1.18 (0.74, 1.88) | 0.97 (0.61, 1.53) | 1.31 (0.77, 2.24) |

Legend text: Data are presented as hazard ratio's with 95% confidence interval for UCC- SMART type 2 diabetes patients with cardiovascular history that were eligible versus patients that were ineligible for each trial.
